# From inception to innovation: bibliometric analysis of the evolution, hotspots, and trends in implantable collamer lens surgery research

**DOI:** 10.3389/fmed.2024.1432780

**Published:** 2024-08-19

**Authors:** Qing Zhang, Di Gong, Kunke Li, Kuanrong Dang, Yun Wang, Changfeng Pan, Zonghui Yan, Weihua Yang

**Affiliations:** ^1^The Third Affiliated Hospital of Xinxiang Medical University, Xinxiang Medical University, Xinxiang, China; ^2^Shenzhen Eye Institute, Shenzhen Eye Hospital, Jinan University, Shenzhen, China

**Keywords:** implantable collamer lens, ICL, refractive surgery, bibliometric, hotspot, trend

## Abstract

**Background:**

As one of several refractive surgeries, Implant Collamer Lens (ICL) surgery offers stable biocompatibility and consistent, high-quality visual outcomes. ICL has become an effective complement to corneal refractive surgery, gradually becoming one of the mainstream methods for correcting refractive errors. This study employs bibliometric methods to analyze research on ICL surgery to understand the progress, hotspots, and potential future trends in this field.

**Methods:**

This study performed a bibliometric analysis of all ICL-related articles collected from the Web of Science Core Collection database between January 1st, 1996, and December 31st, 2023. The CiteSpace 6.2.R4 tool, Excel and the Web of Science website were used to analyze data by country, institution, keywords, and clusters of keywords. Additionally, an in-depth interpretation and analysis were conducted on the field’s high-impact articles.

**Results:**

Since the first clinical application report of ICL, there have been a total of 875 studies. The number of papers published annually has shown an overall increasing trend. Studies published from China are the most numerous, accounting for 29.14% (*n* = 255) of the total. Among the institutions, Fudan University and Kitasato University both have published more than 50 papers, with Kitasato University having the highest H-index of 26. The journals with the top 10 publication volumes are all specialized in ophthalmology. The burst keywords since the introduction of ICL surgery have been “intraocular lens,” “refractive surgery,” and “cataract surgery.” The current burst keywords include “visual quality,” “vector analysis,” “axial length,” etc. The results of keyword clustering included ICL, pIOL, high myopia, axial length, optical quality, refractive surgery, ICL implantation, and pupil size. In the High-impact Articles, it was found that the high-impact articles predominantly focus on the safety, efficacy, and predictability of ICL surgery.

**Conclusion:**

Research on ICL has grown since its clinical introduction, with the advent of the central hole ICL sparking a surge in recent hotspots, particularly in China. Current hotpots in the field of ICL surgery are “visual quality,” “ICL implantation,” “vector analysis,” “axial length,” “evo ICL,” “ICL v4c,” and “ICL.” ICL surgery research trends have evolved from implantation techniques to biological parameters associated with ICL surgery and the benefits of new ICL designs.

## 1 Introduction

Implantable collamer lens (ICL) is a minimally invasive surgical lens implanted in the eye for the correction of refractive errors such as myopia, hyperopia, and astigmatism. The ICL as a Phakic intraocular lens (pIOL) for posterior chamber lenses has been widely used in recent years for the correction of refractive errors (1, 2). Compared to the Artisan IOL (a type of anterior chamber IOL), the ICL has a broader range of applications (including high myopia with or without astigmatism), more stable safety and effectiveness, as well as more advanced model designs (3, 4). Therefore, it has become the market leader in pIOLs. There are four sizes of ICL (12.1 mm, 12.6 mm, 13.2 mm, and 13.7 mm) designed to fit various white-to-white corneal diameters. The selection of these sizes is typically based on specific ranges of white-to-white corneal diameter and anterior chamber depth. The appropriate ICL size is essential for ensuring postoperative vault and reducing the risk of complications (5). This surgical option has become increasingly relevant as the incidence of myopia, particularly high myopia, has risen worldwide (2). Despite its shorter development time, ICL surgery is favored for its wide correction range and effectiveness. Advancements in ICL design, like the central hole ICL, have simplified the procedure by eliminating the need to preoperatively create a hole in the iris (6, 7). The continuous refinement of ICL surgery has led to stable biocompatibility and consistent, high-quality visual outcomes (8).

**Figure 1:**
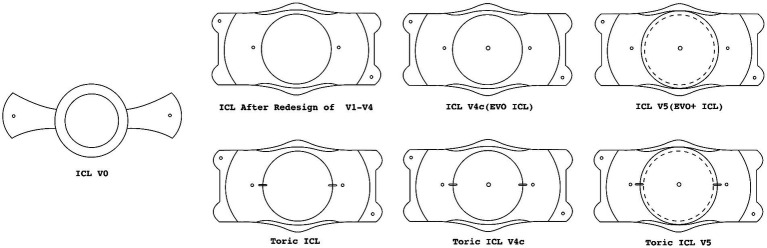
ICL model development. The dotted line represents the ICL V4c optical zone.

The development of ICL surgery ongoing since its inception in 1986, Professor Fyodorov innovated the first collar-stud style posterior chamber intraocular lens, heralding a new era in ophthalmic surgery (9). The groundbreaking design of this lens served as the prototype for the “Visian implantable collamer lens,” subsequently developed by STAAR Surgical Company. The proprietary material used for the ICL surgery, known as “Collamer, “is a combination of methyl methacrylate and porcine collagen, which provides the lens with unique optical properties and biocompatibility (8). The introductory research on ICL surgery was published in 1996, sparking sustained interest and a steady stream of research in the field. Figure 1 has organized schematic diagrams of several classic models used in clinical applications. The original V0 (collar-stud style), during the upgrade process from V1 to V4, gradually perfected the structure of haptics and vault, and expanded the optical zone. The V4c with “Central FLOW” technology, and the subsequent V5 which has a larger optical zone compared to V4c (V4c has an optical zone of 6.2 mm-7.3 mm, while V5 has an optical zone of 6.3 mm-7.6 mm) (10, 11). Toric ICLs have different curvatures on the vertical and horizontal axes, and the added markings can help doctors to position more accurately during surgery, making Toric ICL effective in the treatment of astigmatism (12).

Before the V4c lens, patients receiving ICL surgery models like V4a or V4b needed a YAG laser peripheral iridotomy to ensure postoperative aqueous humor circulation and prevent complications. The V4c lens, with its central hole, has streamlined this process, enhancing aqueous humor flow and reducing the risk of postoperative intraocular pressure elevation (13). Additionally, the central hole promotes natural aqueous humor circulation, potentially lowering the incidence of postoperative cataracts (14–16). Consequently, the V4c lens has contributed to a reduced rate of complications post-ICL implantation (17). Supporting this advancement, Shimizu, K and colleagues have demonstrated through fluid dynamics simulations and the study of aqueous humor flow distribution between the ICL posterior surface and the crystalline lens anterior surface that the central hole ICL can indeed increase the circulation of aqueous humor (18).

Building upon these pivotal developments, the current study meticulously charts the history of ICL surgery research and pinpoints the pivotal hotspots, while elucidating the trends and evolutionary trajectories in this specialized realm since the first clinical trials were documented.

## 2 Materials and methods

Bibliometric tools have been employed to analyze articles on ICL surgery, utilizing graphical and visualization methods to enhance intuitiveness and comprehensibility. This current study harnesses both general and high-impact articles from the ICL academic domain for bibliometric analysis, focusing on contributing countries, institutions, publishing journals, and emerging keywords (19). Through detailed observation of the distribution and trends over the years, including the network of collaborating countries, keyword usage, and research domains within the ICL surgery field, this analysis uncovers development trajectories and potential gaps in the research (20). Visualization of such data enables the identification of focal research areas pursued by institutional teams across different nations, delineating the research domains they contribute to and the intrinsic relationships within the literature. This is instrumental in mapping out the hotspots and providing foresight on future trends in ICL surgery research.

Bibliometric analysis is performed by determining the search strategies and selecting articles. The search strategy includes limiting databases, search terms, language, document type, and publication date. This article uses the Web of Science Core Collection (WoSCC) as the source of bibliometric data. WoSCC encompasses a variety of research journals and provides various bibliometric indicators (such as titles, institutions, countries/regions, publication years, categories, and keywords). To ensure the accuracy and authority of the retrieved data, the indexes chosen are SCI-Expanded (SCIE) and SSCI.

Given the development history of the ICL surgery, the search strategy for this study involves a combination of topic search (TS), title search (TI), abstract search (AB), and keyword plus (KP) within the Web of Science database using a specific set of terms related to ICL. The strategy is as follows: (((TS = (“Implantable Collamer Lens*” OR “ICL” OR “EVO” OR “Visian” OR “Phakic Intraocular Lens*” OR “Phakic IOL”)) OR TI = (“Implantable Collamer Lens*” OR “ICL” OR “EVO” OR “Phakic Intraocular Lens*” OR “Phakic IOL”)) OR AB = (“Implantable Collamer Lens*” OR “ICL” OR “EVO” OR “Phakic Intraocular Lens*” OR “Phakic IOL”)) OR KP = (“Implantable Collamer Lens*” OR “ICL” OR “EVO” OR “Phakic Intraocular Lens*” OR “Phakic IOL”).

The document type has been specified as “Articles,” and the search is confined to the English language. Within the Web of Science database, the “Ophthalmology” category has been selected from “Citation Topics Meso” based on the Leiden algorithm (21), which sorts research publications according to the patterns of citations and references between them. Ophthalmology specialists further exclude articles unrelated to ICL. This method ensures the inclusion of all articles relevant to the field of ICL surgery, spanning the publication dates from January 1, 1996, to December 31, 2023. The objective of adopting this strategy is to encompass a thorough compilation of literature that enriches the understanding of the ICL surgery domain within the stipulated timeframe.

**Figure 2:**
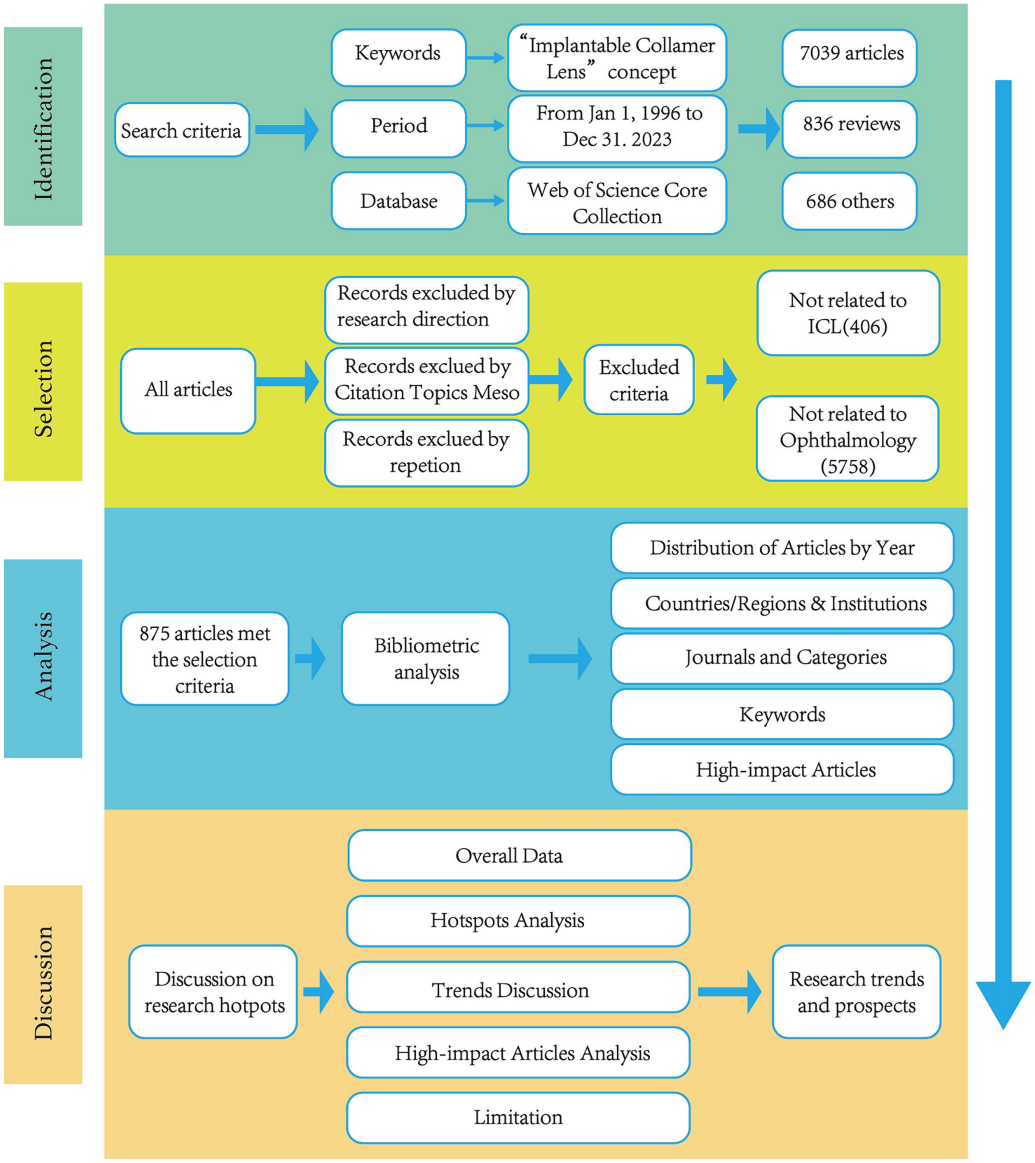
Frame flow diagram.

To ensure accuracy and relevance, two professional researchers specializing in ophthalmology independently performed the data screening. They eliminated records that did not pertain to ICL throughout the search sequence. The subsequent collection of articles was then processed for bibliometric and visualization analyses, with the search and analytical methodology depicted in Figure 2.

To analyze the collaborative networks of countries or regions, institutions, journals, as well as keywords, the study employed CiteSpace 6.2.R4 software and Excel. Concurrently, the WoSCC database’s analysis function served to assess the volume of publications annually and to quantify the contributions per country or region.

## 3 Results

### 3.1 Distribution of articles by year

Over 28 years, the study encompasses 875 articles, collectively cited 16,032 times, including 7,917 self-citations, resulting in an average citation rate of 18.32 per article. The foundational article in this domain was presented in 1996 in the Journal of Cataract and Refractive Surgery, detailing the implantation of a posterior chamber phakic lens composed of collamer to correct myopia in patients. Although the study observed no progression of cataract formation at that stage; it was unable to definitively ascertain the long-term safety of this surgical procedure (22).

**Figure 3:**
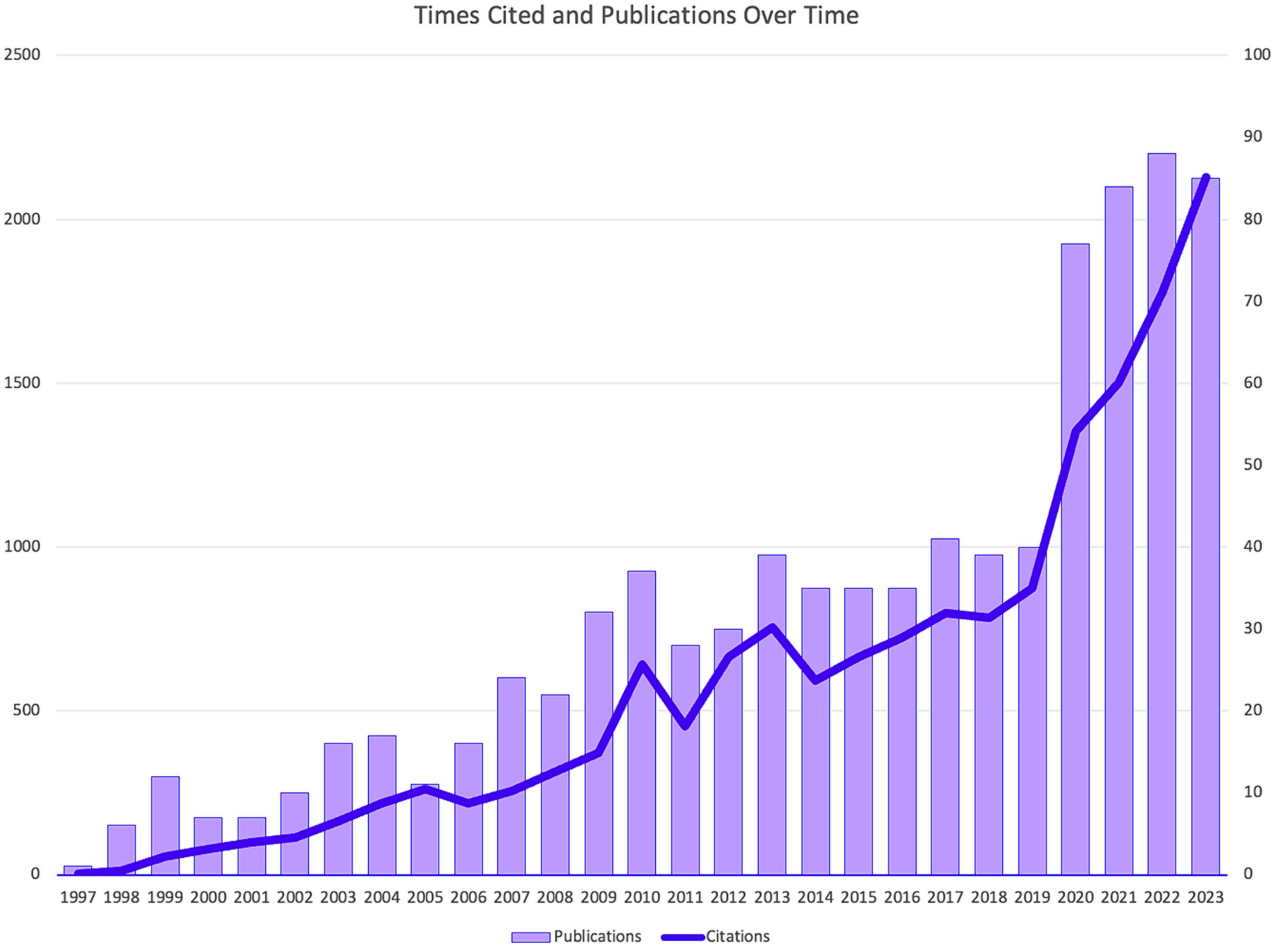
Times cited and publications over time.

Figure 3 illustrates the temporal distribution of publications and citation numbers within the field of ICL surgery research. A sharp increase in the number of published studies is observed between 2019 and 2020, akin to a rocket-like ascent, with a more gradual rise noted in the years following 2020. This rapid increase can be attributed primarily to a surge in research related to the post-operative stability of the corneal endothelial cells in ICL surgery, as well as studies involving “vector analysis” and other parameter analyses. Additionally, the citation trends have mirrored this upward trajectory.

### 3.2 Countries or regions

**Figure 4:**
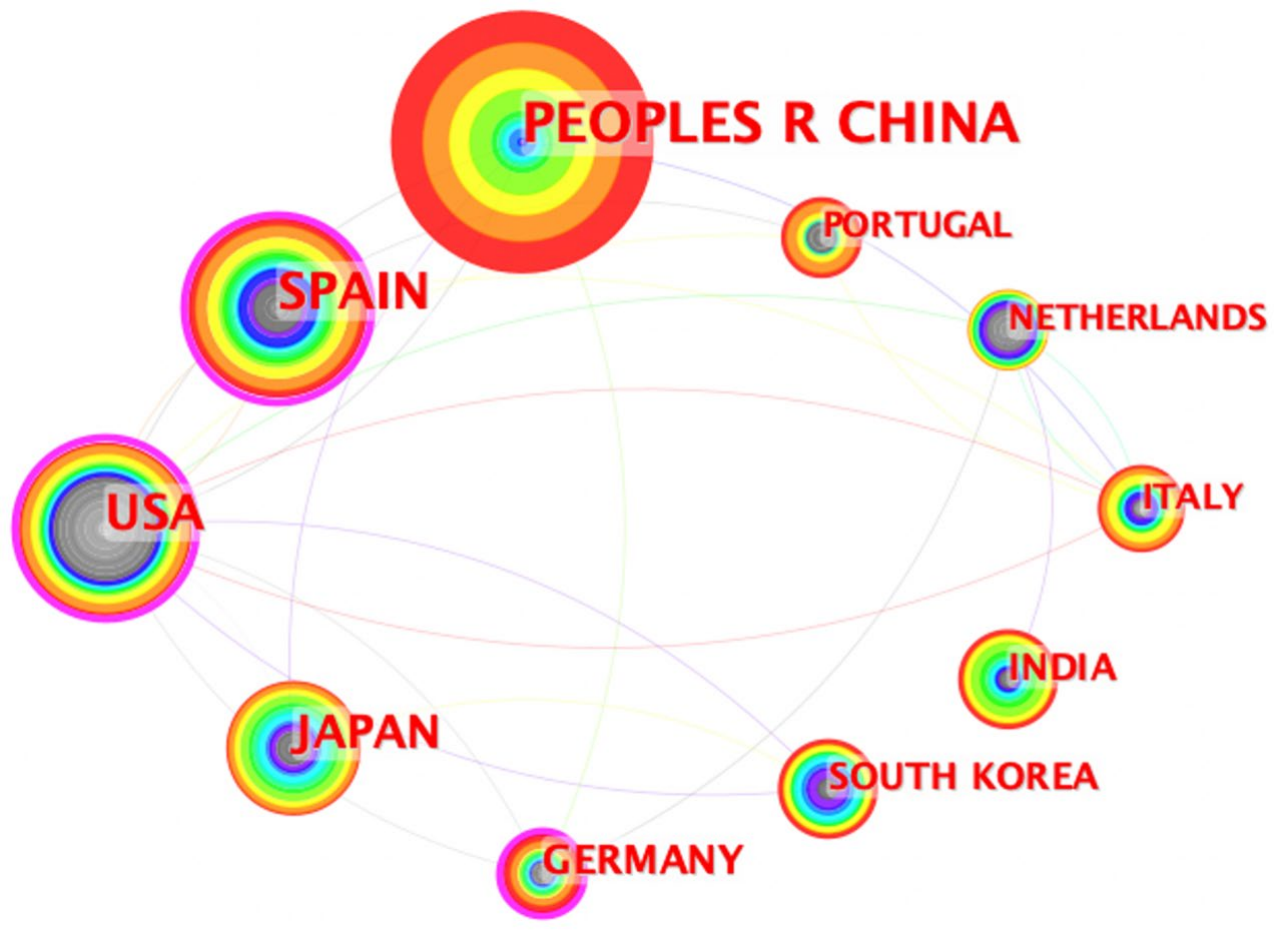
Cooperation of countries or regions.

This study’s articles span across 65 countries and regions. A collaborative network map visualized in Figure 4 indicates the article output of each country, with the size of the labels and nodes being directly proportional to their article count. China, with 255 papers, Spain with 137, and the United States with 124, have the most prominent labels and nodes, signifying their leadership in publication output. Moreover, the density of connections between the nodes signifies the extent of collaboration among these countries or regions; with a denser network reflecting stronger collaborative ties.

Table 1 enumerates the top 10 countries or regions based on their article numbers. It includes their centrality scores, which mirror the intensity of collaboration, alongside H-index values representing their scholarly impact (23). Notably, Spain and the United States exhibit high centrality scores, at 0.50 and 0.51, respectively, coupled with robust H-index values of 33 for Spain and 31 for the United States. China leads the count in articles with 255 entries but has a lower centrality score of 0.03, although it maintains a respectable H-index of 22, indicating substantial influence.

**Table 1:** Top 10 countries or regions with articles on ICL surgery.

Rank	Country or region	Counts	Centrality	H-index
1	China	255	0.03	22
2	Spain	137	0.50	33
3	United States	124	0.51	31
4	Japan	85	0.01	32
5	Germany	47	0.24	17
6	South Korea	34	0.00	19
7	India	34	0.00	11
8	Italy	28	0.05	11
9	Netherlands	27	0.03	13
10	Portugal	26	0.00	14

### 3.3 Institutions

Table 2 presents the top 10 institutions ranked by their research article output, highlighting Fudan University and Kitasato University as leaders with over 50 articles each. Every institution listed in the top 10 has contributed a minimum of 20 articles to the field. Kitasato University stands out with the most significant scholarly impact, boasting an H-index of 26. Geographic analysis of these prominent institutions reveals that four are located in China, four in Japan, one in Spain, and one in Egypt.

**Table 2:** Top 10 Institutions with articles on ICL surgery.

Rank	Institution	Country/Region	Counts	H-index
1	Fudan University	China	70	13
2	Kitasato University	Japan	60	26
3	University of Valencia	Spain	47	20
4	Shanghai Res Ctr Ophthalmol and Optometry	China	41	11
5	Chinese Academy of Medical Sciences and Peking Union Medical College	China	34	6
6	Sanno Hospital	Japan	30	15
7	University of Oviedo	Japan	30	18
8	Nagoya Eye Clinic	Japan	23	11
9	Egyptian Knowledge Bank (EKB)	Egypt	22	10
10	Zhejiang University	China	20	9

### 3.4 Journals and categories

The corpus of this study consists of 875 articles distributed across 92 different journals. Table 3 synthesizes information on the top 10 journals, ranked by their publication volume within this field. In addition to listing the journals, it denotes the percentage of the total publications that they constitute and their calculated H-indexes specific to the discipline. At the forefront, the Journal of Cataract and Refractive Surgery leads with 169 articles, followed by the Journal of Refractive Surgery with 136 articles. These journals hold H-indices of 39 and 34, with impact factors of 2.8 and 2.4 in 2022, respectively. Given the field’s specific focus, all journals featured in the top 10 are exclusively ophthalmology-oriented.

**Table 3:** Top 10 journals with articles on ICL surgery.

Rank	Journal	Record count	% of 875	Impact factor	H-index
1	Journal of Cataract and Refractive Surgery	169	19.3	2.8	39
2	Journal of Refractive Surgery	136	15.5	2.4	34
3	BMC Ophthalmology	55	6.3	2.0	10
4	American Journal of Ophthalmology	46	5.3	4.2	23
5	European Journal of Ophthalmology	31	3.5	1.7	9
6	Graefe’s Archive for Clinical and Experimental Ophthalmology	27	3.1	2.7	13
7	International Journal of Ophthalmology	26	3.0	1.4	8
8	International Ophthalmology	26	3.0	1.6	6
9	Ophthalmology	24	2.7	13.7	19
10	Indian Journal of Ophthalmology	23	2.6	3.1	7

### 3.5 Keywords

**Figure 5:**
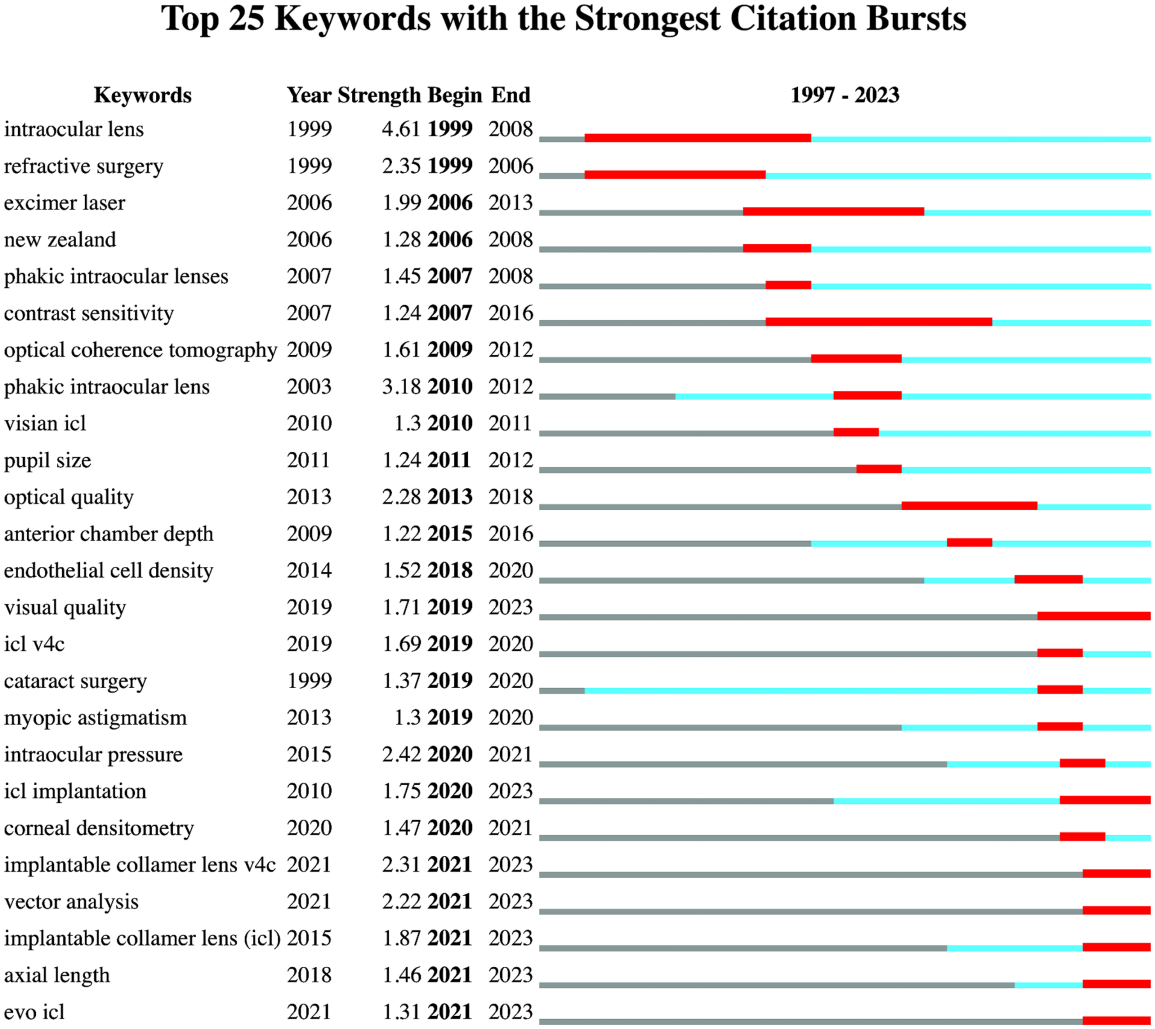
Keywords with the strongest citation bursts.

Employing CiteSpace for co-citation keyword analysis, keywords were extracted from bibliographic entries for text processing and analytical scrutiny, The analysis was configured with a Time span of 1996–2023, a Slice Length of 1 year, and a Selection Criterion of the g-index with k set at 25. Figure 5 vividly showcases the 25 most frequently cited keywords. The intensity of each keyword’s depiction reflects its citation frequency and visibility across the timeframe. Red squares denote periods with noticeable increases in keyword usage. “Active time” is defined as the period during which a keyword has been continuously utilized. Recently emerged keywords are those that appeared in the literature within the last 3 years. An initial keyword burst highlighted terms like “intraocular lens” and “refractive surgery.” Prolonged active use of keywords such as “intraocular lens,” “refractive surgery,” and “cataract surgery” dates back to 1999. Recently, there has been a noticeable uptick in keywords including “visual quality,” “vector analysis,” “axial length,” “evo ICL,” “implantable collamer lens v4c,” and “implantable collamer lens.”

### 3.6 High-impact articles

High-impact articles are significant markers of research impact and are recognized as key achievements within their respective fields. Table 4 lists the 10 articles most frequently cited in the area of ICL surgery research. This list encompasses: pivotal clinical trials on ICL approved by the United States Food and Drug Administration (FDA) (24–26); seminal clinical studies from 1998 evaluating posterior chamber intraocular lenses crafted from collamer material aimed at correcting refractive errors (27, 28); and comprehensive retrospective analyses on ICL interventions for refractive error corrections. These studies collectively measure outcomes such as uncorrected visual acuity (VA), best-corrected visual acuity, adverse events, and both surgical and postsurgical complications, including the extent of lens opacity. Their primary focus lies on assessing the safety, efficacy, and predictability of ICL surgeries. Table 4 also incorporates the limitations identified during these research endeavors.

**Table 4:** High-impact articles.

Rank	Article title and year	Source title	Authors	Cited	Limitation
1	United States Food and Drug Administration clinical trial of the implantable collamer lens (ICL) for moderate to high myopia - Three-year follow-up(2004)	Ophthalmology	Sanders, DR et al.	304	For patients with high myopia, the risks associated with ICL surgery may be increased.
2	US food and drug administration clinical trial of the implantable contact lens for moderate to high myopia(2003)	Ophthalmology	Vukich, JA et al.	208	Correction for severe myopia may face limitations; moreover, there is individual variability within the outcomes.
3	Posterior chamber phakic intraocular lens for myopia of −8 to −19 diopters(1998)	Journal of Refractive Surgery	Zaldivar., R et al.	179	Compared to pIOL, ICL is associated with a higher risk of subsequent cataract formation, and the surgery could be a risk factor for future retinal detachment.
4	Safety of posterior chamber phakic intraocular lenses for the correction of high myopia: Anterior segment changes after posterior chamber phakic intraocular lens implantation(2001)	Ophthalmology	Jiménez-Alfaro, I et al.	158	Postoperative complications following ICL implantation may include loss of endothelial cells and decreased lens transparency, and the long-term contact of ICL with the iris and the natural lens has the potential to cause enduring complications.
5	Eight-Year Follow-up of Posterior Chamber Phakic Intraocular Lens Implantation for Moderate to High Myopia(2014)	American Journal of Ophthalmology	Igarashi, A et al.	155	Myopic regression following ICL implantation may be correlated with factors such as lens nucleus sclerosis, corneal expansion, corneal edema, matrix synthesis, compensatory epithelial proliferation, and axial elongation.
6	Toric implantable collamer lens for moderate to high myopic astigmatism(2007)	Ophthalmology	Sanders, DR et al.	134	/
7	Anterior subcapsular opacities and cataracts 5 years after surgery in the Visian implantable collamer lens FDA trial(2008)	Journal of Refractive Surgery	Sanders, DR	126	There is a certain rate of cataract occurrence in the course of correcting high myopia with ICL, particularly regarding the risk of anterior subcapsular opacities and clinically significant cataracts.
8	Staar Collamer posterior chamber phakic intraocular lens to correct myopia and hyperopia(1998)	Journal of Cataract and Refractive Surgery	Rosen, E; Gore, C	125	There are numerous contraindications to ICL surgery including unhealthy corneal endothelium, abnormal corneal shapes (e.g., keratoconus), lens opacification or incipient cataracts, a history of iritis, glaucoma, pigment dispersion syndrome, lens capsule exfoliation syndrome, and diabetic eye disease. The size of the pupil also has a certain impact on the ICL surgery.
9	Long-term results of implantation of phakic posterior chamber intraocular lenses(2004)	Journal of Cataract and Refractive Surgery	Lackner, B et al.	124	/
10	Four-Year Follow-up of Posterior Chamber Phakic Intraocular Lens Implantation for Moderate to High Myopia(2009)	Archives of Ophthalmology	Kamiya, K et al.	122	The pupil size can potentially influence the ICL surgery to a certain extent, and there are risks associated with cataract development and loss of corneal endothelial cells.

## 4 Discussion

### 4.1 Overall data

ICL surgery, a notable solution for refractive error correction, has seen advancements in material and structural design since its inception. The inceptive ICL was implanted in 1993, representing the first generation of prototypes. Traditional ICL models, preceding the V4c model, necessitated a preoperative laser peripheral iridotomy or an intraoperative iridectomy to avert pupillary block (29). The newer V4c ICL iteration and subsequent models, featuring a central hole, cultivate better aqueous humor flow and preserve central vision (30). An uptick in clinical utilizations of ICL from 1996 to 2023 is highlighted by publication analyses. With ongoing evolution, ICL surgery research hotspots and citations exhibit a growing pattern. Current article trends reveal a peculiar decline in the number of articles in 2023, yet citations continue to ascend. Given the worldwide surge in myopia prevalence, research in this domain is poised for further expansion. Between 2020 and 2023, articles remained robust, averaging over 75 papers annually, which dovetails with China’s significant research output—contributing 178 articles—and broader clinical application of ICL surgery (31).

Aligned with the ongoing progress in ICL surgery, this research delineates not only the trends within the ICL surgery domain but also discerns hotspots, prolific countries/regions, institutions, and keyword clusters. Figure 2 identifies China as the leading contributor in terms of publication volume, albeit with minimal centrality, attributable to the comparatively recent initiation of ICL research in the country. Before 2010, a mere 8 Chinese articles featured in this bibliometric analysis; the subsequent upswing is possibly linked to China’s vast population and the growing prevalence of myopia (32). Spain, the United States, and Japan showcase high H-index scores, signaling an earlier embrace of ICL surgeries and heightened scrutiny in the sector. Analysis of institutions producing prolific research reveals that both China and Japan count four institutions each among the top 10, indicating extensive ICL surgery research activity in these territories. Specialty journals such as the “Journal of Cataract & Refractive Surgery” and the “Journal of Refractive Surgery” lead in published and cited articles numbers. Journals like “American Journal of Ophthalmology” and “Ophthalmology,” despite featuring fewer than 50 articles, still achieve a commendable H-index. Notably, the four mentioned journals are the source of 9 out of the 10 most impactful papers. This suggests that publishing ICL-related research in these established journals could elevate academic prominence and impact for researchers in the field.

### 4.2 Hotspots analysis

In bibliometric studies on keyword bursts and clusters, the centrality of ICL surgery within ophthalmic research is evident. High myopia, optical quality, and pupil size are among the keywords drawing significant attention. This focus reflects the importance of ICL surgery in correcting refractive errors without altering the natural refractive media, which often results in stable or enhanced postoperative visual quality. Furthermore, the anterior or posterior chamber of the eye, due to its implication in the risk of ICL surgery complications, features prominently in current research. This field’s interest is seen in the prevalence of keywords such as “pupil size,” “anterior chamber depth,” “white-to-white,” and “vault” among ophthalmic researchers.

Since the advent of widespread research in this domain, attention to high myopia correction has been prevalent. An early study published in J. Cataract Refract. Surg. in 1996 already evidenced ICL surgery as a viable treatment for high myopia, despite the lack of long-term safety data at that time (22). With the evolution of ICL, various models have emerged for patient selection, alongside alternative refractive surgeries, hence the importance of optical quality comparisons between different ICL models (33) and refractive procedures (34). Research has substantiated that ocular parameters can influence ICL surgery outcomes. For instance, pupil size is critical for lens positioning and impacts surgical success (35). Anterior chamber depth is vital for proper ICL sizing and placement to prevent postoperative complications (36). White-to-white measurements are essential for lens sizing (37) and significantly affect the postoperative vault—the interval between the ICL and the crystalline lens (38). Moreover, this vault is informed by several ocular factors, like white-to-white and anterior chamber depth, which cumulatively determine the prognostic results of ICL procedures.

An analysis of various keywords related to ICL surgery revealed that early research in the field of ICL focused on ICL surgery to evaluate the safety, efficacy, and postoperative refractive outcomes of ICL surgery (39, 40). From the keywords “excimer laser” and “refractive surgery,” we found that many studies were conducted in this field comparing and combining ICL surgery with other refractive surgeries, and in many of these studies, ICL surgery achieved favorable results. In many of these studies, ICL surgery has resulted in good visual quality (41–43). In the study of ICL surgical complications, we found that cataract surgery has been active for a long time and is exploding during 2019–2020, which is associated with the possibility of complications cataracts in ICL surgery (24).

The advent of new ICL variations like the Visian, Toric, and EVO/EVO+ lenses exemplifies the field’s dynamic nature. Distinctive for its central hole, has simplified the procedure by eliminating the need to preoperatively create a hole in the iris (6, 7), the V4c model of ICL encourages aqueous humor flow and cuts down complication risks such as cataracts and pupillary block (44, 45). This design innovation has sparked research interest in recent years. As Figure 5 shows the burst words, “visual quality,” “ICL implantation,” “vector analysis,” “axial length,” “evo ICL,” “ICL v4c,” and “ICL” have become the current hotspots.

The ICL V4c represents a completely redesigned approach compared to previous ICL models, offering numerous benefits. The central hole of the ICL V4c facilitates the natural flow of aqueous humor, maintaining normal intraocular pressure and reducing the risk of postoperative complications such as corneal edema, glaucoma, and cataract. The central hole contributes to the stability of the ICL within the eye, minimizing the potential for rotation or displacement, which is crucial for maintaining the intended refractive outcomes and ensuring the long-term safety of the surgery (46). The central hole in the V4c and subsequent models allows for more precise determination of vault dimensions, which is essential for the effective function and comfort of the ICL. Vault dimensions, as an important postoperative observation indicator, show that the ICL V4c demonstrates good long-term effects and stability (47). In the long-term follow-up after V4c ICL implantation, no significant difference in endothelial cell density was observed between pre-operative and post-operative measurements (47).

### 4.3 Trends discussion

Research within the ICL domain, with a sustained focus on “visual quality,” has ventured into analytical concepts like “vector analysis,” “corneal densitometry,” and “axial length.” Several factors determine the image quality after ICL surgery, including the lens’s optical properties, the stability of its placement, and its effects on axial alignments. Vector analysis, in particular, is crucial for astigmatism management in ICL surgical procedures, enhancing the precision of refractive results and overall visual quality. Its utility spans comparisons across surgical methods and devices, promoting refinements in ICL surgical techniques (48).

Technological advancements in diagnostic tools have steered ICL prognosis studies from broader complications toward a nuanced evaluation of anterior segment metrics. Corneal densitometry, signifying corneal clarity, assists in tracking corneal healing, while “axial length” is instrumental for selecting ICLs (49), forecasting refractive power, and managing surgical risks.

Various measurement devices related to ICL surgery, such as ultrasound biomicroscopy (UBM) and anterior segment optical coherence tomography (AS-OCT), play a significant role in enhancing the precision and safety of ICL surgery. UBM, with its high-resolution ultrasonic imaging, can delve into the structures of the anterior segment of the eye, especially the ciliary body and zonular fibers behind the iris, making it the gold standard for anterior segment imaging. AS-OCT generates high-resolution cross-sectional images of the anterior segment using near-infrared light. Corneal topography is used to assess the shape and regularity of the cornea, and these examinations are crucial for determining patient suitability for ICL surgery and customizing the lens (50). In addition to this, white-to-white corneal diameter measurement is typically conducted using a wavefront aberrometer (Wavelight) or other optical devices to determine the size of the ICL. Assessment of the anterior chamber depth ensures there is adequate space after ICL implantation to prevent contact with the natural lens, thereby reducing the risk of complications (51).

Recent research has focused on developing new methods for ICL size selection based on measurements obtained from these devices. For instance, one study utilized ocular biometric parameters measured with a Heidelberg anterior segment optical coherence tomography device to formulate the optimal lens size, emphasizing the importance of accurate size selection in preventing postoperative complications (50). By integrating these parameters, predictive models related to ICL can be developed to improve the predictability and safety of ICL implantation (52). Furthermore, the integration of advanced measurement tools such as UBM and AS-OCT into ICL surgery has not only improved preoperative assessment but also enhanced the accuracy of ICL size selection, leading to better postoperative outcomes. As these technologies continue to evolve, ophthalmologists gain deeper insights into the structures of the anterior segment, facilitating more precise surgical planning and patient care (51).

Further bibliometric analysis reveals the prominence of specific keywords in the discourse on prognostic ICL surgery research, with “vault, ““central hole,” and “pupillary block” recurring as key concepts. These keywords have been identified with notable frequency, appearing 39, 42, and 12 times, respectively, underscoring their significance in the literature and highlighting their relevance to the prognostic aspects of ICL surgery research. The vault refers to the vertical distance between the posterior surface of the ICL and the anterior surface of the natural lens, which is an important indicator for evaluating the safety of ICL implantation surgery (53). Low vault values risk ICL contact with the crystalline lens, heightening complication odds like cataract development or pigmentary dispersion syndrome. Conversely, excessive vaults can elevate intraocular pressure, potentially causing glaucoma. Because central hole designs in ICLs benefit aqueous humor dynamics and lower elevated intraocular pressure and pupillary block risks, examining the complications associated with new ICL iterations presents a forward-looking research trajectory (54).

The integration of artificial intelligence (AI) in the field of ICL surgery, has led to significant advancements. Machine learning algorithms are now utilized to predict the optimal vault height of phakic IOLs using metrics from AS-OCT, enabling a more precise and personalized assessment of ICL sizing (55). Additionally, AI facilitates the analysis of vast datasets to identify patterns that enhance surgical strategies and predict postoperative outcomes (56). Image recognition through AI allows for detailed evaluation of the anterior segment, ensuring accurate ICL placement. Predictive modeling with AI further assists in foreseeing surgical results, including visual acuity and potential complications, thereby providing surgeons with invaluable insights to make informed decisions (57).

### 4.4 High-impact articles analysis

Upon a thorough review of 10 high-impact articles, it is clear that the safety and long-term prognostication of ICL surgery have consistently been a research priority. In early ICL clinical studies, a demand emerged to refine the power calculation formula, aiming to raise the forecast accuracy for postoperative visual quality (27). Research reporting on the United States FDA’s ICL trials highlighted various adverse events and complications during the trial stages (24, 25). Due to the more recent introduction of ICL surgery relative to other corrective procedures, a dearth of long-term outcome data was noted, prompting newer studies to pursue extended follow-up durations (58). Advancements in research have solidified the understanding of ICL’s safety, efficiency, and outcome predictability. Early ICL surgery research has had a considerable influence on later studies. In the last 5 years, shifting attention has been noticed toward the role of ocular biological measures (59), refining ICL calculation methodologies (60), integrating big data with artificial intelligence for outcome prediction (57), and conducting retrospective analyses with extended follow-up periods (61).

With the innovation of ICL surgical practice, its significant improvement in safety, stability, and outcome predictability has been validated. Looking to the future, as data analytics and algorithmic modeling rapidly advance, the development of nuanced ICL measurement formulas and clinical outcome predictive models stands out as the next research wave in the ICL surgery domain. These innovations are projected to fine-tune surgical planning and enhance the visual results for patients.

### 4.5 Limitation

#### 4.5.1 Limitation of ICL surgery

As an invasive procedure, ICL surgery carries risks of trauma-related complications, including conjunctival or intraocular hemorrhage, corneal epithelial damage, and corneal Descemet’s membrane detachment. Ancillary issues such as anterior or posterior chamber angle injury and traumatic cataract can occur (62). The procedure may also disrupt the ocular surface microenvironment, possibly inducing dry eye syndrome (63). The corneal incision might cause refractive astigmatism, posing a correction challenge (64). Residual viscoelastic substances could obstruct the anterior chamber angle, leading to severe complications like ischemia or permanent ciliary body paralysis (65). Postoperative complications comprise abnormal ICL positioning (dislocation, rotation, inversion) (66), elevated intraocular pressure, and secondary glaucoma (67). Early postoperative cataracts are linked to surgical trauma; later-stage ones are usually due to contact with the natural lens (68). Other common issues include irregular vaulting, corneal endothelium loss, and night vision disturbances (69).

High-impact articles also discuss conditions contraindicating ICL, such as compromised corneal endothelium, irregular corneal morphology, lens opacities or incipient cataracts, a history of iridocyclitis, glaucoma, and pigment dispersion syndrome, all impacting the procedure’s suitability (28).

#### 4.5.2 Limitation of bibliometric

Limitations of bibliometric analyses stem from data selection constraints. Relying on WoSCC data for the SCIE and SSCI indexed articles in the “Ophthalmology” category may result in overlooking seminal works on ICL materials and structure. Such exclusions could skew the representativeness of research cluster findings.

The H-index is influenced by the time of publication and may not fully capture the enduring impact of research, as it does not account for the potential delay in citations or the variability in citation practices across different fields (70).

By only considering articles indexed in SCIE and SSCI and published in English, pivotal studies on ICL material innovation and clinical trials may be omitted. Bibliometrics, while quantitatively robust, focusing on citation counts and publication volumes, may not fully capture the qualitative impact of research. Additionally, the ‘citation lag’ affecting newly published research might not instantaneously represent the true impact of these works.

## 5 Conclusion

Since the first publication in 1996, the field of ICL surgery has shown a year-on-year increasing trend in research output. The surge in publications over the past 3 years may be related to the significant amount of research conducted in China. Among the nations researching ICL surgery, China has the highest number of publications, while Spain, the United States, and Japan have higher H-index scores, indicating their substantial academic impact. Most literature in this field is published in professional ophthalmology journals.

During our research, we identified the following hotspots in the development of ICL: (1) hotspots regarding surgery and complication management, including terms like “ICL,” “refractive surgery,” and “cataract surgery”; (2) biometric parameters related to ICL surgery such as “anterior chamber depth,” “pupil size,” “visual quality,” “vector analysis,” “axial length,” “white-to-white,” and “vault”; (3) novel ICL models like “evo ICL” and “ICL V4c.”

In the trend research of ICL surgery, we have noticed that from the initial exploration of ICL implantation to various biological parameters associated with ICL surgery, and the benefits of new ICL designs in managing postoperative complications. The safety, efficacy, and predictability of ICL surgeries have always been the focal points, as demonstrated by high-impact articles. AI-involved research may be the future trend in the field of ICL surgery.

As an invasive ocular procedure, ICL surgery carries certain risks, including complications related to anterior or posterior chamber trauma. Our research has identified limitations: we analyzed the publication volume and citation counts in the field of ICL surgery from 1996 to 2023, but these metrics may not fully reflect the quality of the literature. Moreover, the latency in citation data may have prevented us from capturing the latest research developments in this field promptly.

## Data Availability

The original contributions presented in the study are included in the article/supplementary material, further inquiries can be directed to the corresponding author/s.
